# Exploring key factors associated with training quality among elite para-athletes. A qualitative study

**DOI:** 10.3389/fspor.2025.1561641

**Published:** 2025-06-09

**Authors:** Vemund Øvstehage, Silvana Bucher Sandbakk, Jan Kocbach, Øyvind Sandbakk

**Affiliations:** ^1^ Centre for Elite Sports Research, Department of Neuromedicine and Movement Science, Norwegian University of Science and Technology, Trondheim, Norway; ^2^ School of Health Sciences, Kristiania University of Applied Sciences, Oslo, Norway; ^3^ School of Sport Science, UiT The Artic University of Norway, Tromsø, Norway

**Keywords:** disability, impairment, paralympics, sport performance, training process

## Abstract

**Introduction:**

Despite extensive research on training load characteristics, knowledge about training quality remains sparse. Training quality encompasses two dimensions: quality in the training process and quality in specific training sessions. Elite para-athletes perform high volumes of training, and their training execution may be further influenced by their impairment and sport. No research has explored training quality among elite para-athletes. Therefore, the aim of this study was to qualitatively explore key factors associated with training quality among elite para-athletes across different impairments and sports.

**Methods:**

A hermeneutic phenomenological approach was applied to conduct semi-structured interviews with 21 Norwegian current and former elite para-athletes (10 women and 11 men) from ten sports and five impairment categories. A reflexive thematic analysis was conducted to inductively explore the experiences and perspectives related to training quality in the training process and specific training sessions. Methodological integrity was ensured through transparency, reflexivity, and member reflection.

**Results:**

Similar overarching themes emerged for the quality of the training process and specific training sessions: (1) Planning and preparation (with themes such as goal setting, recovery strategies, and equipment optimization), (2) Training execution (with themes such as training load management, awareness, and solution-oriented modification of training), and (3) Coaching and training support (with themes such as trustful coach–athlete relationship, individualized interdisciplinary support, and supportive social and professional networks). These themes could influence the quality in each dimension to different extents, depending on the specific impairment and sport, and could either enhance or diminish training quality, depending on how they were addressed.

**Conclusions:**

Based on the experiences of 21 elite para-athletes, this study highlights the importance of planning and preparation, deliberate execution of training, and a competent coaching and support team to influence training quality both in the training process and specific training sessions. The extent to which the underlying factors may impact training quality, depends on impairment and sport. Athletes may improve training quality by making deliberate decisions tailored to their unique prerequisites, goals and resources. Knowledge of these factors can inform para-athletes and coaches in their efforts to enhance quality in the training process and specific training sessions.

## 1 Introduction

Training load characteristics such as training duration, frequency, intensity, mode and associated recovery strategies have been extensively studied in numerous sports (1–4), illustrating the complex interplay of factors depending on the athlete's individual characteristics and sport-specific demands (5–7). Despite the extensive examination of training load characteristics in different sports, knowledge about the quality of training is sparse (8), although training quality is a concept commonly referred to when successful athletes describe why and how they achieved success (8, 9). Hence, there is an unrelieved potential to explore and understand the quality of training (3).

Shell and colleagues (10) previously defined training quality as: “An athlete's capacity to complete a training session to the desired level”. Thereafter, Bucher Sandbakk and colleagues (8) suggested a more nuanced definition: “The degree of excellence related to how the training process or training sessions are executed to optimize adaptations and/or improve overall performance”. In this context, training quality includes at least two dimensions, as it can be examined both from a training process perspective and specifically for the execution of single training sessions (3). By accepting the latter definition, these dimensions of training quality are regarded interconnected and complementary (8), and the study specifically asked the following questions to be explored in future research: “What is training quality?”, “Which factors influence training quality?”, and “Is it possible to assess training quality?”

A training session normally includes a preparation, execution, and recovery phase, where the aim is to provide the bodily system with a specific stimuli, which subsequently may create an adaptive response (8). A session design is aligned with the session's goal and involves a sport specific description including choice of modality, intensity, duration as well as technical and mental focus (1). In this context, Shell and colleagues (10) propose technical, tactical and mental aspects to constitute training quality. McGrath and colleagues (11) found, when interviewing eleven rugby players, that training quality in team sports include team work, and physical, technical, and mental aspects. However, each specific training session is part of a long-lasting training process and thereby considered interrelated with other sessions to create the overall training stimulus.

A training process includes several subsequent sessions orchestrated together to close the gap between the actual and desired capability, thereby inducing long-term development (3). Such processes are often periodized in macro-, meso- and micro-cycles to structure an appropriate pathway to one or multiple performance peaks (12). Commonly, the process is planned and executed in cooperation with a coach and support staff (1), and also includes factors such as recovery, organizational factors surrounding the athletes (e.g., economy, support, access to equipment), environmental factors (e.g., training facilities, gyms), social factors (e.g., team-mates, clubs, family) and health-related conditions (e.g., injury, illness, impairment) that all may influence the quality of the training process (13).

The training process of elite para-athletes normally contains annual volumes of 600–1,100 h, dependent of the individual athlete and sport, in addition to the complexity associated with their inherent impairment (14–16). In this context, it is imperative to understand facilitators and barriers related to their impairment (13). In a holistic perspective, it has been found that the barriers for sport participation and performance among Dutch elite para-athletes were mostly environmental, e.g., dependency of others, transport, equipment, and lack of adapted facilities and qualified supervision, while facilitators tended to be more of a personal character (17). Meanwhile, facilitators and barriers among elite para-athletes have not yet been specifically related to how they influence training quality, and there are also indications that some elite para-athletes do not experience more barriers than non-disabled athletes (17). Accordingly, determination of training quality must be modified to the prevalent characteristics of the athlete, impairment and sport.

The Norwegian Paralympic sport system is integrated into the overall sports organization through the Norwegian Olympic and Paralympic Committee and Confederation of Sports (NIF), where para sports are considered a natural part of the broader sports movement. The system is based on principles of equal opportunity and inclusive practice, providing adapted support for participation at all levels, from grassroots to elite sports, within national federations and clubs (18). In a study conducted within this system, facilitators of long-term sport development of para-athletes were found to be adapted equipment, and a supportive social- and sport network, where sport participation was led by coaches that focused on possibilities rather than limitations (19).

The most represented impairments in the Paralympics are spinal cord injury, limb deficiency, visual impairment, cerebral palsy and muscle deficiency (20). Impairment characteristics may influence the session quality due to for example restricted exercise capacity caused by altered autonomic regulation and reduced active muscle mass, reduced exercise efficiency related to muscle spasticity, decreased range of motion or pain (13, 17, 21–24). Moreover, impairments influencing the movement pattern in daily training and recovery can make athletes more vulnerable to illness and overuse injuries compared to e.g., non-disabled athletes (25, 26).

Due to the heterogeneity among elite para-athletes experiencing the training demands, their unique perspectives and individual experiences on the quality of training would inform and empower coaches and support staff to influence the training process positively (10, 27). Such knowledge about the promoters and inhibitors of training quality may indeed improve the planning, execution and evaluation of training in para-sport.

Rooted in the framework suggested by Bucher Sandbakk and colleagues (8), this study aims to qualitatively explore key factors associated with training quality among elite para-athletes across different impairments and sports. Accordingly, the following research question was asked: What factors do elite para-athletes perceive as most important for training quality?

## 2 Materials and methods

### 2.1 Research design and epistemology

The methodology of in-depth semi-structured interviews with elite para-athletes was chosen to address the aim of this study. To explore the athletes’ individual experiences, perceptions, and reflections on the influence of impairment, as well as the promoters and inhibitors of high training quality, a hermeneutic phenomenological approach (28) was applied throughout planning, data collection, and data analysis processes. As the concept of training quality has not yet been extensively addressed in previous research literature, we applied the definition proposed by Bucher Sandbakk, and colleagues (8) as the conceptual framework: “The degree of excellence related to how the training process or training sessions are executed to optimize adaptations and/or improve overall performance”. Semi-structured interviews were guided by the questions raised in the paper: “What is training quality?”, “Which factors influence training quality?”, and “Is it possible to assess training quality?” (8).

### 2.2 Study participants

#### 2.2.1 Participant recruitment

Athletes were recruited through their respective coaches, support teams, and/or via email and received written information about the project and the extent of their participation. Athletes were selected across different impairments and sports to capture the complexity and comprehensiveness of perspectives within Paralympic sports. All athletes were chosen from within the Norwegian sports system, ensuring a similar overall context to facilitate comparison across impairments and sports. To be included, participants needed to be current or former elite para-athletes with experience competing in the Paralympic Games, World Championships, European Championships and/or World Cup, and between 18 and 65 years old. Furthermore, participants needed to be classified with either spinal cord injury or spina bifida, amputation or congenital limb deficiency, visual impairment, cerebral palsy, or muscle deficiency, because these types of impairments are the most represented in the Paralympic Games (20). Moreover, athletes had to compete in individual para-sports, so that their perspectives on training quality were related to their individual characteristics. Finally, at least 5 years of experience with sport-specific training was required to be considered eligible to provide reflected and insightful experiences on the topic.

#### 2.2.2 Study sample

Twenty-one Norwegian current or former elite para-athletes, from ten different sports and five impairment categories across various regions of Norway, participated in this study. Due to the risk of identification, the number of athletes within each sport and impairment category is omitted. The sample represented an approximately equal gender distribution, comprising 10 women and 11 men, 18 current and 3 former athletes, ranging in age from 19 to 64 years (Table 1). During data collection, some of the athletes were preparing for the Paralympic Summer Games in Paris, while others were training for a regular World Cup season. The number of participants was determined, and recruitment finalized once the experiences relevant to the research question and the richness of information in the interviews were considered sufficient.

**Table 1:** Participants, impairments and sports.

Demographics	
Women (*n*, %)	10.0, 48.0
Men (*n*, %)	11.0, 52.0
Age (mean, ±sd)	33.8 ± 12.0
Years in their main sport (mean ± sd)	17.6 ± 9.0
Age when introduced to sport (mean ± sd)	7.0 ± 5.9
Impairments (*n*): Cerebral palsy, limb deficiency^a^, muscle deficiency, spinal cord injury^b^, visual impairment	5.0
Sports (*n*): Alpine skiing, badminton, biathlon, climbing, cross-country skiing, cycling, rowing, swimming, table-tennis, triathlon	10.0
Sitting sport (*n*, %)	7.0, 33.0
Standing sport (*n*, %)	14.0, 67.0

Data are presented as mean ± sd for continuous variables and *n*, % for count variables.

^a^ Including amputation and congenital limb deficiency.

^b^ Including spina bifida.

### 2.3 Ethical considerations

This research project was approved by the Norwegian Agency for Shared Services in Education and Research (SIKT, ref. nr. 441007) prior to data collection. The participants provided written consent, which included information about the purpose of the research project, participation considerations, risk of identification, data storage, and publication of results, before the study commenced. Information was also provided orally. Participants had the right to cancel the interviews or withdraw from the study at any time.

Due to the low number of para-athletes at the elite level in Norway, there is a high risk of identification. Combining impairment and sport in connection with quotations could lead to the identification of specific athletes. Therefore, our findings are presented with caution regarding the combination of such characteristics. Types of impairments and sports are presented in Table 1, without further details on specific numbers. However, we acknowledge that some sports have only a few athletes at the elite level, which may make an athlete's participation identifiable.

### 2.4 Data collection

The conceptual framework of training quality proposed by Bucher Sandbakk and colleagues (8) was applied to structure the interviews. As this framework divides training quality into two dimensions, the semi-structured interview was *a priori* divided into (a) training quality in the training process and (b) training quality in specific training sessions. The semi-structured interviews began with introductory questions and continued with a similar structure for both dimensions, with minor variations reflecting the characteristics of each dimension. It included questions about: “Factors associated with high training quality,” “How to measure and assess training quality,” and “Potential barriers to high training quality” (8). Examples of questions considering training quality in the specific training session were: “Think of some exceptional training sessions that you have performed. What were the primary factors that were important for attaining good training quality in those sessions?”, “How do you assess or measure the training quality of a session?”, and “Which barriers or obstacles, if any, for training quality in specific sessions, have you identified?” Since this is a part of a larger project, we pilot-tested the interview guide on a para-athlete, para-coach/scientist, and coach for non-disabled athletes prior to data collection. Adjustments following pilot testing involved rewording some questions to enhance clarity.

The interviews were conducted between October 16, 2023, and September 12, 2024, at various locations in Norway. The interviews were conducted 1–3 months ahead of their main competitive season, and none were interviewed right ahead of the Paralympic Games. One semi-structured interview was performed digitally via Microsoft Teams, while the others were conducted in person. The in-person interviews took place in quiet meeting rooms, with the recording device placed on the table between the researcher and the participant. Each interview lasted 60–130 min, with an average duration of 96 min. Interviews began with dimension 1: “Training quality in specific training sessions” (average duration of 53 min), followed by a 5–10 min break, and then continued with dimension 2: “Training quality in the training process” (average duration of 43 min).

To facilitate a smooth start and ease the transition to open-ended questions, interviews began with introductory queries. Predominantly open-ended questions were asked, complemented by occasional closed questions to confirm, paraphrase, or clarify when something was unclear. Participants could request that questions be rephrased or restated. Relevant topics that emerged during the interviews were explored in greater depth through the hermeneutic phenomenological lens. This allowed the researcher to apply previous experience within para-sport to explore the athletes’ lived experiences (28), e.g., important factors for training quality occurring between sessions, or the extensiveness of equipment adaptation. When necessary, participants were asked to contextualize their answers with specific training situations or elaborate further. Athletes were given ample time to reflect throughout the interviews. To ensure a comfortable setting and, where necessary, provide adapted facilities, several interviews were arranged at familiar locations when possible.

The interviews were conducted using an encrypted Dictaphone app, designed and secured for research purposes by Nettskjema (version 4.2.0) (29). The same procedure was followed with a backup device. After the interviews, files were encrypted and securely stored on the Nettskjema platform (University of Oslo), approved for red-level data storage. All interviews were transcribed using an AI-assisted transcription tool in Nettskjema, and anonymized transcripts were downloaded for further analysis. All interviews were conducted and analyzed in Norwegian.

### 2.5 Analysis

#### 2.5.1 Data analytic strategies

A reflexive thematic analysis was performed by the first author (VØ) to, within the two dimensions, inductively explore the depth of the experiences, meanings, and reflections of the elite para-athletes. The analysis followed a “bottom-up” approach and consisted of six stages defined by Braun and Clarke (30). Furthermore, a hermeneutic phenomenological lens was applied, as it allows for an exploration of each para-athlete's lived experiences related to key aspects of training quality, taking into account their impairment and sport. Moreover, it enabled the main author to interpret meaning in combination with his own background, acknowledging and reflecting on preconceptions, and how that subjectivity might influence the interpretation of the findings (28). This ensured both richness and trustworthiness through the analysis (31, 32).

(1), familiarization with the athletes’ experiences related to training quality was achieved by listening to each interview and calibrating them with the word-by-word AI-generated transcripts to ensure verbatim quality. Manual editing was applied if dialects were used, or the transcripts were incorrect. Initial thoughts and impressions of each interview were noted by the first author during this process. (2), all transcripts were uploaded to NVivo version 14.23.2 and coded inductively within the two dimensions. First, the specific training session dimension was coded for each interview, resulting in 386 codes, followed by the training process dimension, which produced 290 codes. Semantic coding was generally used, with latent codes generated when deeper meanings of relevant aspects for training quality needed to be captured, nuanced, and differentiated. (3), initial themes were generated based on the codes that created meaningful concepts related to key factors associated with training quality, resulting in 20 initial themes for the specific training session dimension and 15 initial themes for the training process dimension.

(4) overarching themes and themes within the two dimensions were developed. Themes were created by merging initial themes that encompassed similar topics. When creating overarching themes, themes representing experiences related to certain topics were grouped together. All transcripts were re-read to align the themes with the main content of the raw data, ensuring they represented shared experiences of key factors associated with training quality across the dataset. This iterative process involved editing, merging, or renaming themes to better describe what was commonly perceived as important for training quality in the data. All themes were discussed, reviewed, and refined by the co-authors. This step resulted in three overarching themes and ten themes for the specific training session dimension, and three overarching themes and seven themes for the training process dimension.

(5) overarching themes and themes were named to reflect their association to training quality, ensuring alignment with the research question. Discussions with a colleague experienced in qualitative research, but unfamiliar with the project, contributed broader perspectives to the analysis. This led to a merging of overarching themes across the two dimensions, recognizing that athletes’ experiences encompassed similar overarching themes but addressed different aspects of training quality within each theme. This final tuning of overarching themes for the two dimensions provided the analysis with a comprehensive meaning of how quality in the specific training session and quality in the training process were interrelated, which was then discussed and agreed upon with co-authors. The final analysis resulted in three overarching themes and nine themes in total. (6) The writing process was initiated, with the key factors within overarching themes and themes discussed and fine-tuned with co-authors throughout its development. Findings and selected quotes were formally translated into English and back-translated by the main and co-authors.

#### 2.5.2 Methodological integrity

Transparency was maintained throughout the research process by informing the athletes about the purpose and project background ahead of interviews, and reporting on how the coding was conducted, and themes emerged. An inductive approach was chosen to maintain an open analytic mindset without pre-determined categories. Concurrently, due to the hermeneutic phenomenological lens and the reflexive thematic analysis (28, 30), the first author's prior experiences in (para)sport were acknowledged and applied to strengthen the analysis, particularly in understanding and presenting the practice-oriented meaning of the data. The first author has a background in practicing sports, studying human movement science, and working with elite para-athletes.

As the main author has an active role and meanings are co-constructed within the hermeneutic phenomenological approach (32), reflexivity was maintained by continuously examining one's own beliefs, preconceptions, background, and practices together with the research team. This facilitated validation of data material and ensured reliability. Discussions also enhanced the understanding of the athletes’ lived experiences, as for example differences between tiredness and fatigue among athletes with cerebral palsy, or what accessibility means for wheelchair users during wintertime. Ultimately, although the meaning of data emerges from both the participant and the interpretation of the first author, a final member reflection was offered to calibrate the manuscript with the participants.

Finally, the co-authors, who are also former athletes and have research experience within the Paralympic field, contributed a valuable perspective. None of the co-authors has a disability or has participated in para-sport. Their convenient distance from the interview material allowed them to pose critical questions during steps 4 and 5 of the analysis. This encouraged the main author to extensively explain the key features of the themes and overarching themes, as well as critically consider the data.

## 3 Results

**Figure 1:**
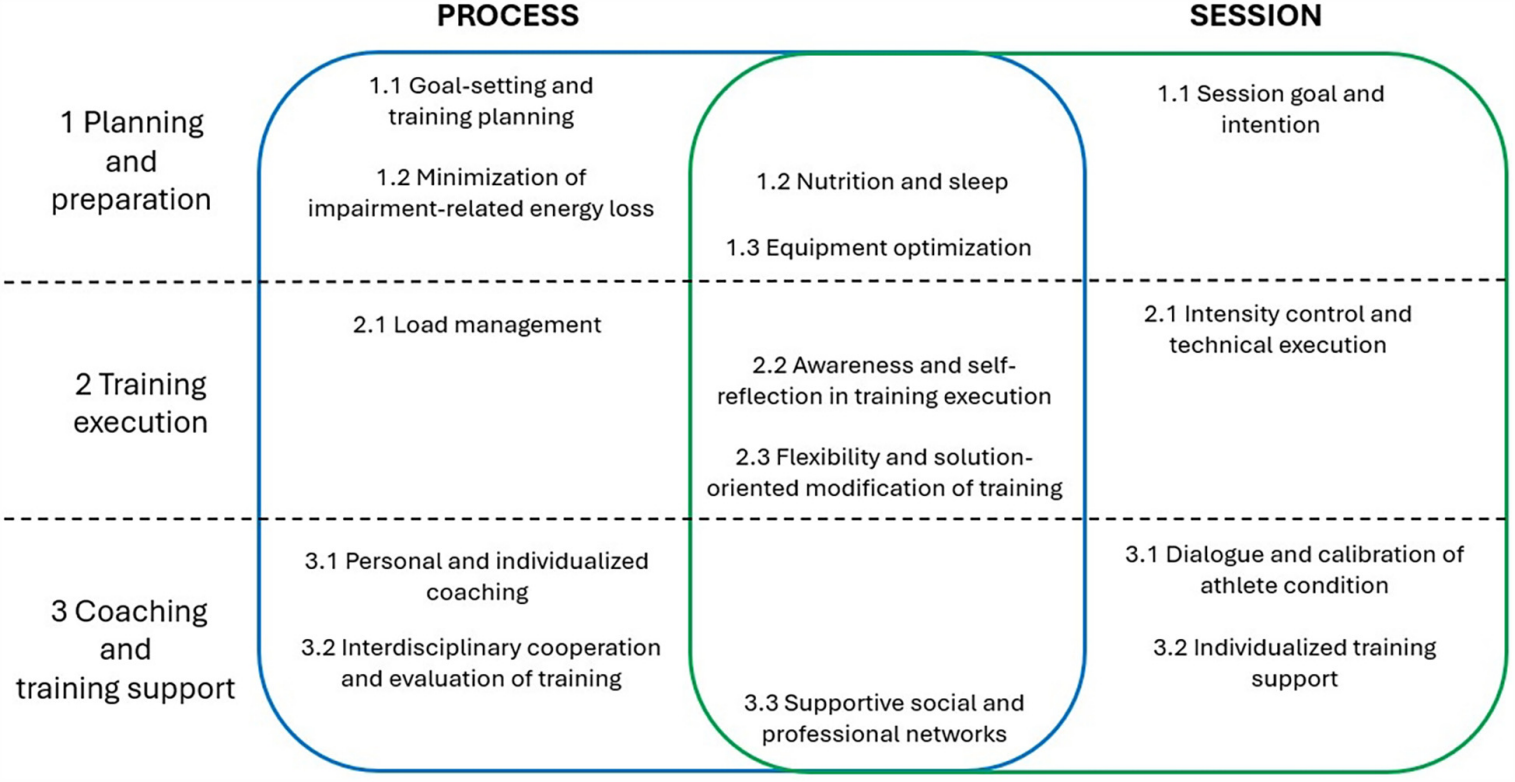
The illustration demonstrates how the overarching themes and themes relate to the quality of the overall training process and specific training sessions. Themes that are critical to both dimensions are positioned centrally, while distinct aspects of other themes are distributed across either one or both dimensions. In latter cases, themes that have impact on both the process and session dimensions have the same number, but wording is slightly different across dimensions.

Three overarching themes and nine themes emerged as key factors influencing training quality in both the overall training process and specific training sessions among elite para-athletes. The overarching themes were Planning and preparation, Training execution, and Coaching and training support. The themes encompassed various aspects of training quality, often specific to particular impairments and sports-specific demands, and could either enhance or hinder training quality depending on how they were addressed. The degree to which each theme influenced the training process and individual sessions is illustrated in Figure 1.

### 3.1 Planning and preparation

#### 3.1.1 Goal setting and training planning to provide training specificity

The overall planning of the training process, along with clear goal setting, was perceived as important for the quality of the process by several athletes (Figure 1). This included periodization of specific training according to the overall goal, as well as structuring session plans based on the goal of the specific period. One athlete pointed out the association between the process and sessions, emphasizing that a session is only “one brick in a bigger wall”: “*To have a clear understanding of the session goal in advance, and that the session clearly is a part of a bigger plan. [...] To make sure that what you do is in line with the overall goal.”* Furthermore, the importance of predictability and long-term planning was underscored by several athletes as beneficial for process quality: “*Structure is very important for me. It helps me to have a clear and agreed plan that has been developed ahead of time. When I know what is coming, I can plan accordingly.”* Several athletes highlighted the degree of certainty and predictability related to training camps, income, work, equipment, or sponsors as important determinants for good quality in a long-term training process. Further, they stated that having such organizational factors unresolved could cause reduced training quality: “*[I notice that this issue] negatively affects recovery, preparation for the next session, and such things. It doesn*’*t matter if the training quality is good when there are so many surrounding issues.”*

Goal setting was perceived to be fundamental for mental preparation by many athletes, who experienced increased training quality by focusing on the specific session tasks. Many endurance athletes expressed that high-intensity sessions required mental preparation, while, for example, table-tennis players emphasized fast-playing and technical sessions as those requiring sufficient mental focus. In this context, goal setting was perceived to enhance session quality if the goal was considered relevant for competition. As exemplified by a swimmer, such goals ensured specificity in the movement patterns: “*You can conduct very hard training, but if you do not execute movements in a speed that the competition requires, you will not practice what you aim to improve.”* A cross-country skier underscored that session goals with high competition relevance required sufficient preparation, which in turn improved the likelihood of high session quality: “*For example, a session where I often perceive that the quality is high could be an 8* *×* *8 uphill interval session that has a set intensity, and where the preparation for it and mindset going in is similar to competition. I start preparing for this session one to two days in advance”.* On the other hand, not having a clear session goal, and thus not knowing what to mentally prepare for, were experienced as characteristics of poor-quality sessions, as noted by a climber: *“The bad sessions are those without a goal, and they have poor focus.”*

#### 3.1.2 Recovery strategies to minimize impairment-related energy loss

A consensus among the athletes was the importance of a structured process to balance the necessity for recovery in order to achieve good training quality. All the athletes pointed at food and sleep as the key components between sessions in a training process. The need for recovery was also linked to their different impairments, and it was highlighted how they needed to plan to succeed with recovery strategies. One athlete with cerebral palsy emphasized how sufficient sleep, food, and recovery were highly associated with good training quality: “*[Training quality] is heavily dependent on the planning before the session to make sure I get enough sleep, food and recovery.”* One athlete also emphasized fatigue as a personal reason why predictability was an advantage for good process quality: “*Fatigue is something I must consider in everyday life, and it is not enough to simply relax an hour before training*.”

Further, the sitting athletes expressed a common experience regarding the difficulty of balancing the total strain on the upper body. Being unable to use their legs for transport and activities of daily living affects the recovery of their upper body. One athlete illustrated the need to be cautious due to overload: “*I have less opportunity to let my upper body recover, which means I have to be extra cautious that I don*’*t overload it.”* Moreover, sitting athletes experienced that energy loss due to colder temperatures, snow, and poor preparation of facilities affected their physical and mental readiness ahead of sessions, possibly reducing training quality: “*When you, in addition, spend more energy on activities of daily living than an athlete without a disability, you start running on empty.”* The accessibility and adaptation of facilities could positively and negatively influence the session quality, as for example the location and state of the parking spots at wintertime, location of toilets in the facility, and the adaptation of gyms.

All the visually impaired athletes expressed the importance of initiatives to save energy and reduce stress throughout the training process. They explained how everyday life tasks were perceived as big energy losses due to their impairment, possibly having a negative influence on their training quality. One athlete illustrated the extensive time and energy consumption of having a visual impairment, saying: “*It is like having a 30% employment as a visually impaired person”.* Finding strategies and establishing good routines were therefore highlighted as important to counteract the lack of independence and avoid logistical stress that could lead to unnecessary energy loss. One visually impaired athlete said: “*[Visual impairment] is a typical factor that means that you are not as independent as others. One example is the additional stress of just going to the grocery store. It is then important not to let yourself get too stressed, and just accept the situation.”* Besides having deliberate strategies of daily duties, another preparation initiative was to move permanently closer to suitable facilities to meet the training demands. Hence, visually impaired athletes emphasized the consideration of facilities as important for good session quality. Delving into this, examples were provided on how light conditions, the number of people in the facility, and facility structure could affect the quality of the session. One athlete said: “*If the sun is shining on the bottom of the pool, then I will get blinded. If there is too little light, I will have night blindness. But, if it is just a little light, then I know my direction in the pool.”*

#### 3.1.3 Equipment optimization

Adaptation and maintenance of equipment were experienced as prerequisites for a successful training process. Sitting athletes and athletes with a limb deficiency considered the fit of their equipment to be crucial. One athlete underscored the individual uniqueness by comparing it to wearing shoes: “*It is very individual, and you immediately feel if something is not fitting right. Especially when practicing sport”.* Another athlete emphasized how the prosthesis could facilitate or attenuate training quality depending on the fit, and, together with other athletes, highlighted the risk of blisters, pain, and overload: “*Optimizing the fit of the prosthesis automatically improves the quality of the session. [A poorly fitted prosthesis] may be painful, and you do not completely trust it, which is discouraging*.”

Due to the uniqueness of the equipment, some athletes illustrated their vulnerability when the equipment was damaged. They described an extensive process of total equipment repair or component replacement, potentially leading to decreased quality in the training process. Athletes highlighted that regular equipment is easier to replace and exemplified the complexity of having customized equipment repaired: “*If [my equipment] is broken, I have to contact a civil engineer and orthopedic engineer, organize the transportation of the equipment to wherever they are, and make sure I can finance the repair. As a consequence, I have to restructure my training plan since I cannot execute it without it [equipment].”* Moreover, a cyclist and a triathlete underscored that the fit of equipment is essential for performance and that there is no chance of buying a new bike in a regular sports store, with the price for a new bike estimated to be 13,000 dollars. One said: “*If I do not have this exact [equipment], I am lost. I have spent many months adjusting it to fit my specific needs.”*

Sitting athletes expressed how the sitting position affects the movement pattern and the ability to create power, which was the main reason for adapting equipment fit. Some of them underscored the marginal adjustments: *[The position was not good.] My leg support was closer than usual, placing my body in a two-centimeter higher position, which resulted in a totally new movement pattern.”* Due to some athletes’ reduced sensitivity in the lower body, challenges were experienced related to colder temperatures and seating position. They described how this could affect performance and build frustration, further decreasing training quality: “*One day I noticed that I could not maintain good technique in the uphill. Turns out my posture was off, but because I cannot feel my buttocks, I did not understand what was wrong.”*

### 3.2 Training execution

#### 3.2.1 Intensity control and technical execution to manage training load

Individual intensity control during sessions was, according to many athletes, a determinant of good training quality for each session. A cross-country skier emphasized the importance of conducting sessions according to intention, independent of type of session, stating: “*Intensity control is probably the most important aspect. It is important to be able to hit and maintain the intended intensity zone, so that you do not go too hard. This also applies to the low-intensity sessions”*.

Many athletes shared this view and expressed concerns about how intensity control in individual sessions affected their total weekly load. They further attributed this particular focus on intensity control to their impairment. A swimmer explained the need to be cautious in order to achieve good process quality: “*It is important to balance individual sessions against the load of the rest of the week. Because of the impairment, I have to be extra mindful that a single session does not cost too much”.* However, varying experiences emerged across sports regarding intensity regulation. Visually impaired athletes described how they calibrated their own perceptions with their guide's impressions, adjusting the intensity accordingly. A cross-country skier, on the other hand, provided an example of how heart rate and previous experiences were used to calibrate session intensity, while one swimmer reflected on how the impairment primarily affected intensity control, highlighting the importance of continuous decision-making during a session: *“I swim slower than the others [non-disabled] in my training group, and it is sometimes tempting to try to keep up with them. But from a training quality perspective, it is important that I keep to my intended pace. I can keep up with them if I want to, but that requires a higher intensity, so I need to be mindful not to do so.”*

Athletes across all sports recognized that awareness and focus during sessions were closely related to training quality. Athletes in cross-country skiing, alpine skiing, and table tennis emphasized that focus was critical for executing good technique during a session. A cross-country skier explained: “*I always have a ‘technique checklist’ in my head to make sure that I do not forget something or develop a bad movement pattern. So, for me, the technical and mental aspects are closely connected.”* In this context, skiers with different impairments underscored the importance of good snow and light conditions for proper technique execution. Climbers explained how the combination of intensity and technique alternated depending on the type of session. They noted that low-intensity sessions were predominantly focused on precise movements during longer climbs, whereas high-intensity sessions involved higher power and shorter boulders that required increased focus. For these sessions, it was essential to execute good technique on every route according to the planned intensity, without hesitation.

#### 3.2.2 Awareness and self-reflection in training execution

Training quality was perceived to be influenced by awareness and self-reflection in both the training process and specific training sessions. Many athletes noted that a clear understanding of training plans, overall goals, working tasks, and how to adjust these components was essential for increasing training quality through self-reflection. An athlete expressed this importance as follows: “*When you know the why and how of what you are going to do and have the right mindset with continuous reflection of how well you meet the intention, [it has a positive effect on training quality].”* Several athletes aimed for a positive mental state to achieve high-quality sessions, which was facilitated through personal commitment and sense of ownership of the training process. On the other hand, a table-tennis player reflected on why lack of ownership hindered self-progression: “*[…] I was just doing what I was told or whatever the other athletes were doing without reflecting over why. While you can still develop with this mindset, you lose ownership of your development, and it becomes difficult to perceive that progression occurs.”*

Awareness of the risk of medical issues also emerged as a significant factor influencing training quality. These medical issues were typically impairment-related and were mainly discussed by sitting athletes. They described challenges with training continuity due to frequent interruption of sessions or longer time out of training during a process. One athlete highlighted how colder temperatures during sessions could have serious consequences: “*You are more vulnerable, and it is important to frequently go to the toilet. Kidney infection is not fun at all, so having individual strategies to reduce the risk of illness and injury is important.”* Protective initiatives and alternative solutions were highlighted as central aspects of good training quality, along with awareness and acceptance of the medical issues that can arise due to their impairments.

#### 3.2.3 Flexibility and solution-oriented modification of training

Flexibility was expressed by most of the athletes as the key to maintaining continuity in the training process. This included restructuring the weekly plan or modifying specific sessions when necessary. Many athletes emphasized the importance of pursuing improvements and maintaining quality despite adjustments to alternative training. Hence, they sustained a continuous drive for success through a solution-oriented mindset, as illustrated by a sit cross-country-skier: “*There is room for alternative sessions compared to the general training plan, especially in our situation. We are very different [from each other], and thus we have different challenges to solve. Therefore, the key is to be solution-oriented, creative, and adaptable, instead of saying: ‘No, I’ll just skip this session, it does not fit me’.”*

Athletes across different impairments noted how assessing their daily condition helped prioritize training quality in sessions. They adopted a broader process perspective and restructured their training plans, thereby achieving better quality in individual sessions. One cyclist explained: “*It is all about the acceptance of adjusting due to present conditions. [...] For example, one day I was supposed to do an interval session, but I decided not to do it that day. Instead, I postponed it to the day after, rested that evening, and did the session with good quality the next morning.”* In contrast, some sitting athletes also shared experiences of poor-quality sessions by describing instances where negative signs were ignored: “*I am not sure you can call it obsessive thoughts, but a need to follow what is planned. […] It is a difficulty in seeing the big picture when I feel I have to execute the session, whatever it takes.”*

Some athletes with limb or muscle deficiencies reported experiencing few barriers to training quality. They often leveraged their individual circumstances to address potential challenges through a combination of solution-oriented thinking and specific adaptations. In this context, these barriers were reframed as motivational challenges that propelled the training process forward: “*I am so used to living with my impairment, so I consider it only as a challenge. I want to make the most out of the situation to be as good as I can.”*

### 3.3 Coaching and training support

#### 3.3.1 Trustful and predictable coach-athlete relationship

Several athletes described the coach-athlete relationship as the cornerstone of training quality. A relationship built on trust, where athletes could openly share their thoughts with the coach, was seen to positively influence the training process. A sitting athlete highlighted this relationship as essential for facilitating individualization and safety in sessions, if dealing with invisible medical impairment-related challenges that required openness and trust to share: “*There may be some invisible issues for para-athletes that you cannot always control. Therefore, it is important to dare to share, instead of training with pain. [...] This is basically connected to a trustful relationship with the coach.”* Without such a relationship, minor issues could turn into major problems, that further could decrease training quality both in the short- and long term. Furthermore, the coach-athlete relationship was experienced as an interplay based on shared goals. Common goals were considered crucial for avoiding misinterpretations between the athlete and coach, fostering discussions on the same level, and creating a sense of mutual dependency. Some athletes described their relationship with their coach as indispensable and as the key factor for achieving high training quality. One athlete stated: “*Training quality is an interplay between the coach and athlete. ‘What do the coach and athlete expect?’ It includes having an open dialogue and trust in each other, like a special connection, to further work on specific goals together.”*

Despite the demands for elite-level quality and performance, many athletes expressed appreciation for a communication climate that allowed open dialogue and honest discussions. One athlete emphasized how their relationship with the coach facilitated constructive discussions, ultimately improving cooperation and session quality: “*The coach and I had to solve some initial problems concerning the training programs because it felt wrong to me. It was crucial for our cooperation. Now, after some years, it works fine: The coach is planning, then we discuss the content before starting, and if necessary, we make adjustments as we go.”*

The determination and confidence of the coach were regarded as fundamental for ensuring good training quality. Moreover, clear communication from the coach was described as relieving, especially as many athletes must consider various impairment-related aspects. A swimmer explained how a confident coach made training easier: “*The point is, you need to know when to listen to your mind and when to listen to your body. You must consider if you are going to adjust according to the performance in training or if you should follow the plan. I have asked the coach to change the plans because I go so slow! Then it is relieving that the coach is consistent and trusts the plan.”*

Most athletes shared experiences of no structured individual assessment of session quality. Instead, they emphasized that quality assessment was primarily conducted through self-reflection related to their perceived sense of accomplishment. However, some athletes described valuable debriefs with their coach, where they summarized the positive and negative aspects of each session. These debriefs, while not directly assessing session quality, were still perceived as beneficial: “*We are assessing mostly tactical and technical aspects, not the direct quality of the session. But we have a dialogue during or after the session, where we consider the focus of the session and what we have done.”*

#### 3.3.2 Interdisciplinary cooperation and individual training support

An interdisciplinary team engaged in the training process was experienced by all athletes as facilitative for maintaining quality in the training process. Many athletes emphasized that impairment-specific knowledge among team members and individualized approaches tailored to specific impairments were crucial for ensuring training quality across all para-athletes. Concurrently, they appreciated the facilitative role of the support team. One sitting athlete expressed the following: “*The individualization and dialogue with every single athlete is extremely important for me as a para-athlete to get the most out of my training. The support team works with different athletes with various impairments that may have different needs.”*

Moreover, it was noted that many para support teams consisted of non-disabled people, which could occasionally lead to misunderstandings and difficulties in fully grasping the consequences of an impairment. One athlete highlighted these challenges with the following statement: “*If I say apple, then they [the support team] need to know how it tastes. [...] They need to understand the consequences of what I am saying. [...] Like my coach, he knows what it demands for me to shuffle snow. [...] Additionally, I guess it is harder for a person that has always been disabled,* *versus* *a person that can compare it to being non-disabled.”* The athlete further underscored the importance of interdisciplinary teams continuously enhancing their competence and emphasized that sufficient para-specific expertise was essential for both the team's development and the quality of training.

Along with the competence of the support team, many sitting and visually impaired athletes illustrated the value of having a support team to facilitate and enhance the quality of session execution. Sitting athletes primarily linked this to equipment facilitation and logistics due to their limited mobility, saying: “*In interval sessions in long hills, it is crucial that I have a person that can drive me back down after the session. The roller-ski sledge has no brakes.”* Visually impaired athletes highlighted the importance of their support team for successful session execution, explaining how their guide or supportive coach ensured the quality of both technical and endurance sessions in unfamiliar facilities or outdoor activities, by e.g., physical movement-based technique instructions, navigation in unfamiliar terrain, or verbal description of surroundings.

Testing as a systematic method for providing feedback was an appreciated evaluation tool for many athletes. They described how testing was frequently used to calibrate the process quality with their coach and support team, especially after a training camp, training period, or tournament. Testing was experienced as both helpful and motivational, as it demonstrated the value of their dedicated training efforts. One cross-country skier explained this motivation as follows: “*We have often had testing after periods with a certain focus, to verify how it worked out. [...] It is fine to see that what you have worked on had a good effect. It is important for my motivation to see that my work has value and to see that I improve.”*

#### 3.3.3 Supportive social and professional networks

Support and clarification at home were perceived by the more experienced athletes as prerequisites for long-term process quality. They explained the value of involving their family as part of their team to maintain the mental and physical capacity required for sustaining high training volumes. It was emphasized that family understanding of the commitments involved in elite sports acted as a foundation for success. One athlete ensured process quality by framing the sport as a family project, with approval from everyone: “*I am dependent on having my family with me on the team to handle the bigger picture. It is important so that I can do what I do and for my quality to be as good as possible in both the session and process. [...] It is like a mutual project.”* Regarding the quality of session execution, clarity and support at home helped reduce stress levels for the athletes, enabling greater focus on session tasks.

Some athletes elaborated on the interplay between training quality and financial concerns in elite para-sports, particularly regarding sponsorship opportunities. Similarly, as exemplified as a prerequisite for process quality in theme 1.1, one athlete explained how securing sponsorship posed a significant challenge for para-athletes and how this directly affected training quality due to the necessity of spending more time working: “*The opportunities for having sponsors and financial support are way lower [than for non-disabled athletes]. Consequently, you must work more because you need to have a way of earning money.”* As highlighted, fewer sponsors and lower income required more time in regular employment, which diverted energy away from sport-specific tasks and negatively impacted training quality: “*I think many athletes find themselves in the same situation, because we do not live off the sport, and therefore, we have to spend our time and energy on other things besides the sport.”* For athletes who needed to work, goodwill from their employer was emphasized as critical. One athlete described the generosity of their workplace and how they provided better prerequisites for Paralympics preparations: “*The boss told me to go all in towards the next Paralympics, and that I could skip my work. ‘You do not have to apply for a leave of absence either. You will get paid.’ [...] That was a great message to receive, which allowed me to dedicate my energy solely to performing.”*

## 4 Discussion

This is the first study to explore the experiences of elite para-athletes regarding what they perceive as important factors associated with training quality. Interestingly, we found similar overarching themes for training quality when considering both the training process and the specific training sessions. For both dimensions, athletes identified planning and preparation, training execution, and coach and training support as overarching themes influencing their training quality. Within each overarching theme, we identified several themes related to the quality of the training process, specific training sessions, or both dimensions. However, the magnitude of influence of each theme appeared to depend on the particular dimension, and characteristics of the impairment and sport.

Among the three questions posed by Bucher Sandbakk and colleagues (8), this study explores the second one, “Which factors influence training quality?”, thereby contributing to the development of training quality frameworks in future research. As suggested by Bucher Sandbakk and colleagues (8), training quality includes two interconnected and complementary dimensions: the training process and the specific training sessions. However, in the interviews, it is notable that the athletes are more reflective on the perspectives of the session. The reason for this may be, as discussed in previous studies (8, 10), that athletes have a natural ownership of the session because they can directly influence the quality of its execution. Moreover, it appears that quality in the training process is experienced as originating from similar overarching themes as those for the session but applied over a longer time frame, being broader and more extensive, and done in a multifaceted cooperation.

The athletes’ experiences in this study suggest that they may enter either a positive or negative cycle affecting their training quality. For instance, a negative cycle is often seen to arise when it is not financially viable to pursue a full-time career as an elite para-athlete, which is the case for many para-athletes due to limited income from sponsorships. This financial strain forces them to take on work or study commitments, in some cases with unfavorable impairment preconditions, which can increase stress and logistical challenges. Consequently, this may exacerbate impairment-related energy loss, reducing the time and resources available for preparation and recovery. As a result, training quality may decline, both in specific sessions and across the broader training process. On the other hand, coaches, support teams, and social or professional networks can act as crucial game-changers, intervening to transform the cycle into a positive chain of events. Such cycles may also be evident for non-disabled athletes, but by drawing this comparison, it can be argued that elite para-athletes with certain impairments may benefit even more from organization of training, transportation to the facility, preparation of equipment, and execution of specific segments of training. Coaches, support-team and surrounding networks that provide tailored support, can enhance training quality from both a process and session perspective. Thus, these two dimensions—process and session—appear interdependent, with one often serving as the input or output of the other (8).

### 4.1 Planning and preparation

The importance of goal setting, recovery routines, and optimization of equipment was perceived to have a pronounced connection to training quality, as well as to the athletes’ impairment and sport. By conducting sufficient periodization and session planning, athletes were able to adjust their recovery strategies and equipment depending on the current session goal and intention. Impaired movement ability may lead to time-consuming processes before and after training, thereby introducing a pronounced need for sufficient predictability and individual adjustments to achieve quality and training adaptations. Elite Paralympic athletes have previously identified time management as a significant challenge for sport participation, particularly when balancing a busy schedule (33). Similarly, our findings highlight the value of structure and predictability for training quality. Thus, planning and preparation appear to support effective time management within the training process.

Goal setting was a method to address performance demands and, accordingly, to identify which performance gaps needed to be filled in the training process. The subsequent elaboration of competition-relevant session goals was experienced as motivational and contributed to maintaining correct focus during sessions, while process goals provided direction. This aligns with the experiences of elite para-athletes preparing for the Paralympic Games in Rio de Janeiro, who perceived their goals as facilitative for staying strongly focused and motivated in everyday training (33). These findings underscore the importance of a deliberate planning phase to achieve high-quality outcomes throughout the training process. Moreover, athletes emphasized the value of mental preparation procedures tailored to specific session tasks, particularly for sessions with high intensity or technical demands. Similarly, the findings of Shell and colleagues (10) highlighted the, technical, tactical, and mental training objectives as clear components of training quality. Thus, the intention of each session emerges as a determining factor for how athletes prepare for and ensure training quality.

For sitting and visually impaired athletes, predictability contributed to better execution of training, as it allowed them to prepare according to the prevalent conditions of the facility, session type, and location. More specifically, the accessibility and design of sport venues, both outdoors and indoors, were experienced as potential barriers to training quality. This aligns with the findings of McLoughlin and colleagues (33), where such issues were identified as challenges to participation among elite para-sitting athletes. However, contrary to our findings, Dutch Paralympic sitting athletes referred to the number of sport facilities in their neighborhood as the main barrier to sport participation (17). A possible explanation for why the athletes in our study did not express similar challenges could be their preparation strategy, which involved relocating closer to proper training facilities. Additionally, previous research suggests that being dependent on others for exercise and transport were primary barriers to exercise among visually impaired athletes (34). This was also relevant for the athletes in our study, who experienced energy loss due to logistical stress and frustration. They addressed these barriers through meticulous preparation, which was critical for facilitating training quality.

Recovery was emphasized as decisive in ensuring training quality and achieving sufficient training adaptations throughout the process. Therefore, strategies such as adequate sleep and proper nutrition routines were considered essential by all athletes, regardless of impairment or sport. A deliberate nutrition strategy was also highlighted by Steffen and colleagues (35) who found gastrointestinal problems to be the second most burdensome issue among Paralympic athletes, typically arising from nutritional shortcomings, including among others, sub-optimal meal-timing as a potential trigger for underlying health challenges related to the impairment. Moreover, in a physical perspective, sitting athletes had to manage their monotonic upper-body load to minimize the risk of shoulder injuries. Such risks have also been highlighted in other studies (36, 37), and thus, particular attention should be given to upper body load for wheelchair users between sessions, as it may further influence training quality. Similarly, athletes with visual impairment or cerebral palsy reported positive experiences with adjusting energy consumption on days with demanding sessions, as also emphasized by Webborn & Van de Vliet (36). Therefore, well-established nutrition routines and impairment-specific recovery strategies appear to be beneficial for training quality, both from a pre- and post-session perspective.

Equipment optimization, particularly impairment-specific adaptations, was emphasized by many athletes as a fundamental prerequisite for training quality. Within the training process, it included injury prevention and the maintenance of functionality, while in training sessions, it involved optimizing movement patterns and power output. Equipment such as bikes, sit-skis, wheelchairs, and prostheses were all subjects to a customized fitting process tailored to the athletes’ bodily functions, restrictions, and dimensions. A previous case study on a sitting athlete demonstrated that adjustments to equipment configurations can lead to significant performance improvements, such as higher peak forces and increased trunk motion (38). However, in some cases, the best adaptations for performance may come at the expense of comfort or a sustainable movement pattern. Athletes in our study elaborated on the challenge of finding prostheses with the optimal combination of fit and function, tailored to their impairment. This challenge is likely due to musculoskeletal compensation mechanisms that influence technique execution, necessitating that both the athlete and their equipment must be considered from a mutual perspective when aiming to improve training quality (39).

### 4.2 Training execution

Training execution emerged as an overarching theme, encompassing the psychophysiological processes influencing the quality of both the training process and the training sessions. Awareness of how to control the training execution, self-reflection, and a solution-oriented mindset for adjusting session characteristics were experienced as substantially important. This can be regarded as training smartness, which contributes to the regulation of internal and external load, both in the short and long term. As previously discussed by Haugen and colleagues (3), training smartness was proposed to be one of the athlete-dependent characteristics that could influence training quality. Correspondingly, Bucher Sandbakk and colleagues (8) emphasized the importance of awareness and focus on planned session tasks, along with continuous control and micro-adjustments of training intensity during the sessions. Additionally, they made connections to the significance of mental preparations ahead of sessions. Improving training quality is, therefore, arguably based on a combination of physiological and psychological aspects that are necessary for conducting adjustments in training execution.

Awareness of why you execute training as planned and how you are going to do it were highlighted as prerequisites for appropriate intensity control and self-reflection in training sessions. The concept of the why and how of training execution was also emphasized by Solli and colleagues (9), and appears to be an essential aspect to understand for intensity control to obtain good training quality. Furthermore, technical execution was perceived to be related to intensity control in our study. Endurance athletes experienced external components, such as terrain, conditions, light, weather, and equipment, as influencing technique execution, making intensity control a complex and challenging task. Hence, this complexity sometimes caused athletes to allow the intensity to increase in order to facilitate the maintenance of proper technique. Correspondingly, Tønnesen and colleagues (1) found that athletes in biathlon, cross-country skiing, and long-distance running increased the intensity from zone 1 to zone 2 in uphill sections to maintain and practice correct technique. In contrast, in swimming, rowing, and speed skating, training in zone 2 was sometimes deliberately applied to develop specific technical aspects. This underscores the importance of awareness when balancing technique execution and intensity control, as athletes sometimes need to prioritize one over the other to execute the session as intended, thereby increasing training quality.

Recognition and acceptance of one's own daily physical condition were experienced as difficult by some athletes, influencing how they adjusted intensity and load. This supports the findings of Foster, Heimann (40), who investigated the perceptions of training among competitive athletes. They found a mismatch between the intended and executed intensity, resulting in athletes going too hard on planned recovery sessions and too easy on harder days. Over the long term, this may affect the ability to manage training load, which may attenuate development. However, the athletes in our study underscored the importance of continuous self-reflection, as well as dialogue with the coach and support team, to succeed in adjusting intensity and load. Likewise, Seiler (4) suggested that a standardized training intensity “language” may close the gap between intensity prescription and execution. Beneficial adjustments in intensity may contribute to continuity and training load management, thus increasing the quality of both the session and the process.

Flexibility in modality, type, and order of training sessions was commonly perceived as a key in the quality of the training process. This solution-oriented mindset allowed for adaptation to conditions that arose, often related to the impairment. Steffen and colleagues (35) found athletes with neurological impairments, predominantly sitting athletes, to be more exposed to illness than those with musculoskeletal impairments. This aligns with experiences in our study, where some athletes acknowledged that medical issues were a part of the game, but where alternative training in close dialogue with coach was a strategy to maintain continuity and quality in training.

An impression from the interviews was that athletes with more experience at the highest level tended to be more reflective and curious in exploring open questions related to their training execution, compared with athletes with less experience at the highest level. Although at least five years of experience in their main sport was an inclusion criterion in this study, the number of years at the highest level could be shorter. Thus, it is arguable that the extent of experience may be related to the level of awareness and self-reflection in training execution. The ability to visualize oneself from different perspectives may be beneficial for detecting elements of training quality that could be improved.

### 4.3 Coaching and training support

Experiences that emerged within this topic point at the necessity of having the organizational factors present surrounding the elite para-athletes, which includes i.e., financial prerequisites for pursuing para-sport at a professional level, high quality support from coach and support team, balanced family and working conditions, and access to equipment and facilities. A trustful coach-athlete relationship, including personal dialogue and honest performance-enhancing discussions based on mutual respect, was considered a fundamental component of training quality. This valuable role of the coach aligns with other studies that emphasize interpersonal closeness and commitment (41). Moreover, successful Olympic athletes have pointed out feelings of trust, respect, and interdependence as central characteristics of the coach-athlete relationship (42). Correspondingly, in previous studies, the coach has been found to play a major role in the training process of elite para-athletes (13), with Canadian athletes, for example, appreciating coaches who were supportive on a personal level (43). These findings align with those of this current study, where many athletes underscored the importance of closeness in their relationship with the coach. Due to the complex combination of elite training and impairment characteristics, it is arguable that a close and trustful coach-athlete relationship is beneficial in many cases within the paralympic field. In this context, the coach's ability to individually adapt the components of the training process seems to be key for the athlete's training quality.

Coaches’ sport-specific knowledge and feedback have been suggested as facilitative for athletes’ performance and success (33). Furthermore, research has shown that elite para-athletes emphasize the benefits of having a coach who can transfer sport-specific knowledge and experience to the relevant para-perspectives (43, 44), which is consistent with our findings where the coach and support team were valuable in customization of training. As illustrated by one athlete in our study, it is not sufficient to know how an apple looks; the coach and support team also need to know how the apple tastes. These underlying impairment-related aspects, earlier referred to as “invisible issues,” require a coach and support team to have knowledge about the impairments of the individual athlete (44, 45). For example, this may concern movement restrictions, impaired muscle activation, medical challenges, or fatigue (36). Interestingly, the majority of para-support teams consist of able-bodied people, which may introduce some challenges in understanding how “the apple tastes” (45). However, for visually impaired athletes in our study, their guide was considered a crucial part of the support team, providing feedback when training in the field. Previous findings in climbing confirm the athletes’ dependency on their guide to execute a route efficiently (46), while in running, sighted guiding runners were considered decisive for high-quality execution by visually impaired runners (47). Thus, providing feedback related to the execution of technical aspects, considering the impairment-specific influence, may enhance training quality.

Supportive social and professional networks, such as practical, emotional, and/or financial contributions from family and employers, emerged as fundamental for training quality throughout the training process. Previously, family support has been shown to ensure well-being and positively influence performance among elite para-athletes (17, 34, 48, 49). For some athletes in our study, family support established a cohesive team feeling and reduced potential stress related to bad conscience. On the other hand, experienced athletes also expressed challenges related to financial balance, requiring additional jobs, studies, and/or sponsorship to create the necessary circumstances for process quality. This was also discussed by Alexandre and colleagues (44), who noted that Paralympic athletes had to engage in dual careers due to the high costs of participating in parasport. In our study, this could be linked to i.e., the high costs of adapted competitive equipment. However, obtaining sponsorship to resolve financial imbalance was regarded as a high-effort, low-reward relationship. This aligns with the experiences of Spanish paralympic women, who experienced fewer opportunities to secure sponsors, likely due to limited recognition of success and media attention (49). In other words, it appears that some elite para-athletes must spend significant amounts of energy on acquiring sufficient funding for an elite-sports career, negatively influencing the quality of the process. Therefore, support from social and professional networks can provide stability that may be a game-changer in reducing stress and enhancing training quality.

One topic that did not extensively emerge was the importance of team cohesion and training with teammates. Except for swimmers and table tennis players who touched on this link to training quality, none of the other athletes elaborated on the effect of the team. This is somewhat surprising, given previous findings where Aitchison et al. (48) found that elite para-swimmers who experienced strong team bonds found their sessions more enjoyable and were more motivated to push harder. Moreover, the team spirit had the potential to positively influence performance. On the other hand, this is also reasonable, as all the sports included in our study were individual sports. However, it could be that the main author's line of questioning was focused on the individual perspectives due to the characteristics of the sport, thereby influencing athletes’ responses. Additionally, one could assume that the different impairments within a team may introduce some challenges in maintaining similar external and internal load, making training together more difficult. Finally, swimming and table tennis are sports that facilitate conducting sessions together due to the fixed indoor area where the activity takes place, which may increase interaction during sessions.

### 4.4 Methodological rigor and limitations

This study interviewed a relatively high number of elite para-athletes, especially when considering the low number of elite para-athletes in Norway, which provides rich and unique data. One of the strengths is the heterogeneity of geographical locations, sports, and impairments, which includes a wide range of experiences of training quality within the paralympic field. However, there are only a few athletes with similar impairments, which also vary within each sport. Thus, the risk of identification had to be addressed by providing limited information in the quotations.

The conduction of interviews in person strengthened the richness of the data, through obtaining a personal and informal dialogue. Furthermore, the extensiveness and depth of the interviews were dependent on the athletes’ ability to reflect upon their own training quality. Here, semi-structured interviews in person combined with a hermeneutic phenomenological approach facilitated better conditions for the thorough exploration of relevant topics, allowing the athletes to provide nuanced experiences and the main author to further delve into relevant topics. However, such an approach allows for co-construction of meanings through interpretation of lived experiences. Hence, trustworthiness was ensured through continuous reflexive discussions with co-workers as well as member reflections of the manuscript (32). Ethical approval and respectful methods were applied to maintain interdependency between the athletes and the main researcher.

## 5 Conclusions and practical implications

Elite para-athletes typically engage in high training volumes while managing individual impairment-related challenges alongside external and organizational factors that may influence both health and performance. Based on the experiences of 21 elite-level para-athletes across various sports, three overarching themes—Planning and Preparation, Training Execution, and Coaching and Training Support—emerged as key determinants of training quality, both in the overall training process and in specific sessions. These factors may impact the two dimensions of training quality differently, depending on the athlete's impairment and sport-specific characteristics.

Elite para-athletes can enhance training quality by making deliberate, individualized decisions based on their unique physical prerequisites, specific goals, and resources. In the short term, high-quality training can optimize session performance, facilitating effective athletic development within the range of possibilities. This aligns with the long-term goal of maintaining a sustainable balance between training load and recovery tailored to the individual para-athlete, thereby promoting health and fostering optimal training adaptations.

Understanding these factors provides valuable guidance for elite para-athletes and their coaches in improving training quality at all stages—before, during, and after sessions. Future research should explore the perspectives of elite para-coaches, non-disabled athletes, and coaches of non-disabled athletes to identify the critical factors they associate with training quality.

## Data Availability

The datasets presented in this article are not readily available because content from interviews containing identifiable information cannot be shared. Requests to access the datasets should be directed to Vemund Øvstehage, vemund.ovstehage@ntnu.no.
